# Clinical perspectives on the age-related increase of immunosuppressive activity

**DOI:** 10.1007/s00109-022-02193-4

**Published:** 2022-04-06

**Authors:** Antero Salminen

**Affiliations:** Department of Neurology, Institute of Clinical Medicine, University of Eastern Finland, P.O. Box 1627, FI-70211 Kuopio, Finland

**Keywords:** Ageing, Immune tolerance, Meta-inflammation, Photoaging, Post-resolution, SASP

## Abstract

The aging process is associated with a remodeling of the immune system involving chronic low-grade inflammation and a gradual decline in the function of the immune system. These processes are also called inflammaging and immunosenescence. The age-related immune remodeling is associated with many clinical changes, e.g., risk for cancers and chronic infections increases, whereas the efficiency of vaccination and immunotherapy declines with aging. On the other hand, there is convincing evidence that chronic inflammatory states promote the premature aging process. The inflammation associated with aging or chronic inflammatory conditions stimulates a counteracting immunosuppression which protects tissues from excessive inflammatory injuries but promotes immunosenescence. Immunosuppression is a driving force in tumors and chronic infections and it also induces the tolerance to vaccination and immunotherapies. Immunosuppressive cells, e.g., myeloid-derived suppressor cells (MDSC), regulatory T cells (Treg), and type M2 macrophages, have a crucial role in tumorigenesis and chronic infections as well as in the tolerance to vaccination and immunotherapies. Interestingly, there is substantial evidence that inflammaging is also associated with an increased immunosuppressive activity, e.g., upregulation of immunosuppressive cells and anti-inflammatory cytokines. Given that both the aging and chronic inflammatory states involve the activation of immunosuppression and immunosenescence, this might explain why aging is a risk factor for tumorigenesis and chronic inflammatory states and conversely, chronic inflammatory insults promote the premature aging process in humans.

## Introduction

**Fig. 1:**
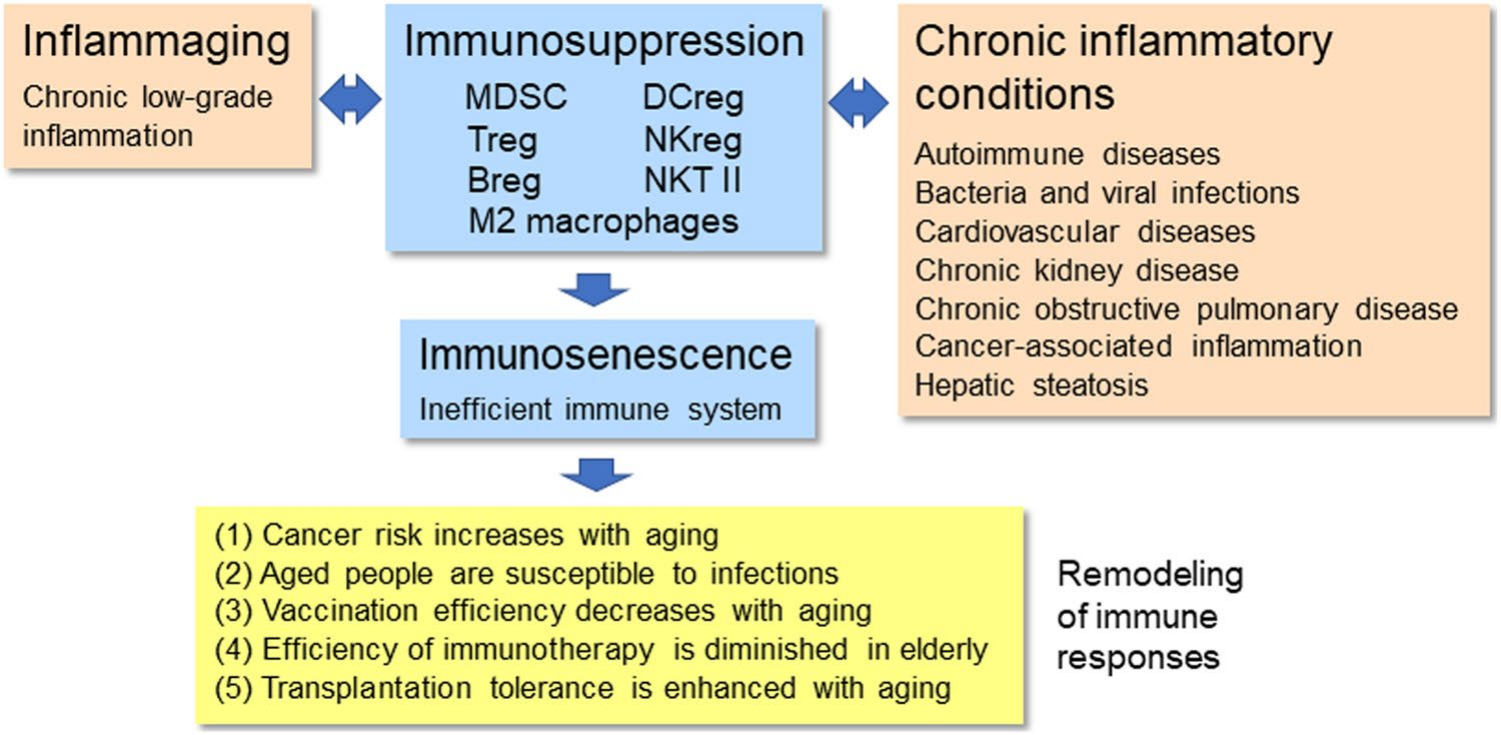
Inflammaging and chronic inflammatory conditions remodel the immune system by inducing a counteracting immunosuppression which promotes immunosenescence. The immunosuppressive network involves MDSCs, Tregs, Bregs, DCregs, NKregs, NKT II cells, and type M2 macrophages. Subsequently, immunosuppression and an inefficient immune system disturb the efficacy of immune responses which increase the risk for cancers and chronic infections as well as decreasing the efficiency of vaccination and immunotherapy. Conversely, the increased immunosuppression associated with aging enhances transplantation tolerance. Abbreviation: Breg, regulatory B cell; DCreg, regulatory dendritic cell; MDSC, myeloid-derived suppressor cell; NKreg, regulatory natural killer cell; NKT II, type II natural killer T cell; Treg, regulatory T cell

The aging process is associated with a remodeling of the immune system in humans and different animal species [1, 2]. The senescence of both immune and non-immune cells evokes a low-grade pro-inflammatory state, called the senescence-associated secretory phenotype (SASP) [3]. There is substantial evidence that the age-related remodeling of the immune system induces many clinical changes, e.g., the risk for cancers and chronic infections increases, whereas the efficiency of vaccination and immunotherapy decreases with aging (Fig. 1). Moreover, it is known that chronic inflammatory states promote the aging process (see below). The resolution phases of inflammatory insults are associated with counteracting immunosuppressive responses which are intended to protect tissues from excessive inflammatory injuries although immunosuppression also promotes the senescence of immune and non-immune cells [4, 5]. Consequently, this provokes many age-related clinical alterations described below. There is convincing evidence that the activation of immunosuppressive network, e.g., myeloid-derived suppressor cells (MDSC), regulatory T cells (Treg), and type M2 macrophages, has a crucial role in tumorigenesis, chronic infections, and the inefficiencies of vaccination and immunotherapy (see below). It seems that the activation of immunosuppressive network promotes the age-related immunosenescence which enhances the age-related clinical immune changes. Here, it will be examined the role of the age-related remodeling of the immune system in the generation of clinically observed immune alterations with aging. Original and review articles were searched from major databases including PubMed, Scopus, and Google Scholar.

## Clinical interactions between aging and chronic inflammatory conditions

### Aging is a major risk factor for chronic inflammatory diseases

There is extensive evidence that aging is a major risk factor for a wide variety of chronic diseases, especially for chronic inflammatory diseases. Given that the aging process is associated with a low-grade inflammatory state [1, 2], it is not surprising that chronic inflammation is involved in many diseases across the lifespan [6]. For instance, chronic inflammation prevails in cardiovascular diseases [7], chronic kidney diseases [8], and neurodegenerative diseases [9]. Moreover, chronic inflammation has a crucial role in the pathogenesis of many cancers [10], the incidence of which significantly increases with aging [11]. Commonly, multimorbidity and frailty exist in the elderly and mortality increases exponentially in aged-related diseases [12, 13]. Several studies have revealed that inflammatory markers predict frailty status and mortality in older people [14, 15].

Centenarians are living examples of a successful aging process and thus these people have been studied in attempts to understand the mechanisms underpinning healthspan regulation. A survey of several community-based prospective studies indicated that the inflammation score significantly predicted the all-cause mortality, capability, and cognition in individuals of extreme old age [16]. Interestingly, the inflammation index of centenarians’ offspring was lower than that of their age-matched controls. Rubino et al. [17] demonstrated in their study on the offspring of Sicilian centenarians that these individuals were more resistant to the aging-induced immune changes than their counterparts. For instance, the serum level of Tregs and γδ T cells as well as that of senescent T cells were significantly lower in the offspring of centenarians as compared to those values of their non-centenarian counterparts. There are other studies indicating that the offspring of centenarian people have a lower prevalence of several age-related diseases [18]. These results imply that epigenetic factors regulate the healthspan of centenarians and their offspring. The epigenetic clock based on DNA methylation has been used to evaluate the biological aging process [19]. Horwath et al. [20] reported that the DNA methylation level of peripheral blood mononuclear cells (PBMC) from Italian semi-supercentenarians and their offspring revealed a decreased epigenetic age compared to the age-matched controls. Currently, the epigenetic regulation of the healthspan and lifespan needs to be clarified. Interestingly, Jylhävä et al. [21] demonstrated that the mortality-associated methylation sites mapped to genes functionally clustering around the nuclear factor κB (NF-κB) complexes in nonagenarians. NF-κB transcription factor is a major driver of inflammatory responses. There is abundant evidence that NF-κB signaling seems to be the molecular culprit of inflammaging process controlling both healthspan and lifespan [22]. It seems that the age-related inflammatory state predisposes an organism to chronic inflammatory diseases which are also driven by the activation of NF-κB signaling.

### Chronic inflammatory disorders promote premature aging in humans

There is convincing evidence that chronic inflammatory insults experienced during childhood or adulthood can enhance the premature aging process in humans. There are examples of both local and systemic inflammatory states which can promote the aging process. For instance, the UV-radiation (UVR)-induced photoaging of the skin is a well-known example of an accelerated aging process driven by repeated inflammatory insults [23, 24]. Recent studies have revealed that UVR and environmental pollution trigger damages in the skin, e.g., oxidative stress and DNA-damage, which evoke a cellular senescent state in skin fibroblasts and keratinocytes [25, 26]. Senescent cells, which possess an SASP phenotype, are secretory cells releasing many inflammatory factors, e.g., cytokines, chemokines, and metalloproteinases. A senescent state is common in aging tissues promoting the aging process and also age-related diseases [27]. Tumorigenesis is another example of an increased chronic inflammatory state which can promote both local and systemic premature aging processes in cancer survivors [28, 29]. The cellular senescence and inflammation occurring in tumor sites enhance the tumorigenesis process. Several epidemiological studies have revealed that either cancer itself or cancer therapies accelerate the biological aging process, e.g., osteoporosis, muscle atrophy, pulmonary fibrosis, cardiotoxicity, and characteristics of frailty [28, 30]. Moreover, cancer survivors suffer from impaired wound healing, increased infections, decreased cognitive status, chronic fatigue, and increased morbidity. These properties indicate that cancers promote premature aging in cancer-survivors.

It is known that chronic infections enhance immunosenescence and are able to promote the aging process in humans (Fig. 1). Cytomegalovirus (CMV) infections increase with aging and in fact, most elderly people are CMV-seropositive. However, CMVs are normally living in a dormant state in the host cells but their reactivation triggers a chronic low-grade inflammation. Several investigators have revealed that CMV infection is able to enhance immunosenescence [31, 32] although this topic is still a matter of some debate. Recently, Hassouneh et al. [33] demonstrated that there are clear differences in the immunosenescent states induced by the aging process or CMV infection, e.g., in alterations of the T cell subsets. Genetic studies have revealed that CMV infection accelerated the epigenetic aging profile of human PBMC cells [34, 35]. Given that CMV infection affects the properties of immune cells and impairs immune responses, it is not surprising that it increases the risk for age-related diseases [31, 36]. The immunosenescence induced by CMV and the ageing process seem to induce a more severe COVID-19 disease in elderly people [37]. There are studies indicating that patients suffering from human immunodeficiency virus (HIV) infection display a premature onset of age-related morbidities, e.g., cardiovascular diseases, osteoporosis, cognitive impairments, and frailty [38, 39]. Gianesin et al. [40] demonstrated that a significant premature aging and immunosenescent phenotype were evident in HIV-infected children. The telomeres of PBMCs were considerably shorter in the HIV-infected than uninfected children. There appeared significant changes in T cell population, e.g., lower thymic output and reduced maturation level of T cell receptors (TCR). It is noteworthy that these alterations were not attributed to antiretroviral therapy, instead these changes indicated the presence of a premature immunosenescent state in the HIV-infected children [40].

There are several other chronic inflammatory states which enhance immunosenescence and promote a premature aging process, e.g., chronic kidney disease (CKD) [8, 41], chronic obstructive pulmonary disease (COPD) [42], and rheumatoid arthritis [43] (Fig. 1). It is known that CKD triggers a persistent uremic inflammation which promotes the appearance of the immunosenescent phenotype involving the loss of thymic function, a decrease in the numbers of naïve B and T cells, the expansion of memory T cell population, and a skewed TCR repertoire [44]. CKD also exposes the individual to the co-morbidity of many age-related diseases. The pathogenesis of CKD involves a typical array of inflammatory changes, such as oxidative stress, mitochondrial dysfunction, and cellular senescence [8]. One specific indicator of CDK is a major decline in the production of α-klotho protein, a well-known anti-aging protein [45]. The reduced secretion of klotho protein from kidney disturbs cellular calcium/phosphate metabolism, thus enhancing co-morbidities, such as atherosclerosis, osteoporosis, and skin atrophy. Klotho protein has many anti-inflammatory properties which confer protection against the immune senescence of human monocytic leukemia cell line-1 (THP-1) [46]. Klotho-deficient mice display many pathological properties which are similar to those appearing during the aging process [47]. Given that the expression of klotho decreases with aging, it seems that the decline in the expression of klotho protein in CKD promotes premature aging processes in the whole body. COPD is also associated with an increase in the hallmarks of aging, e.g., epigenetic changes, telomere shortening, cellular senescence, immunosenescence, and low-grade inflammation [42]. COPD is also accompanied by several co-morbidities. In conclusion, it seems that the persistent inflammatory state, either in the aging process itself or in inflammatory diseases, enhances an accelerated aging process with the appearance of more and more co-morbidities with aging. The mechanisms underlying the inflammation-driven aging process need to be clarified. It is known that chronic inflammation triggers a counteracting activation of the immunosuppressive network which not only induces immunosenescence but also increases cellular senescence in inflamed tissues and in the whole body (see below).

### Inflammation stimulates immunosuppression

Acute inflammatory states stimulate compensatory anti-inflammatory responses which counteract excessive inflammatory reactions, promote resolution processes, and thus prevent unneeded damages to the host tissues [48, 49]. In chronic inflammation, immunosuppressive activity has a crucial role in the stabilisation of inflamed microenvironment. Inflammatory mediators, such as colony-stimulating factors (CSF), chemokines (e.g., CCL2), and interferons, stimulate the expansion of the myelopoietic lineage and augment the generation of myeloid cells in the bone marrow (BM). Increased myelopoiesis stimulates the generation of immature myeloid-derived suppressor cells (MDSC), both monocytic M-MDSCs and polymorphonuclear PMN-MDSCs, which are released from the BM and are subsequently recruited into extramedullary immune sites, e.g., spleen and lymph nodes, or directly into inflamed tissues [50, 51]. In the target sites, MDSCs proliferate and enhance their immunosuppressive potentials to suppress pro-inflammatory responses. The immune cells are exceedingly plastid and the phenotype of immune cells can be modulated from a pro-inflammatory to an immunosuppressive state, a process also called polarization [52]. For instance, monocytes and granulocytes can be converted into immunosuppressive M-MDSCs and PMN-MDSCs in an environment where there is persistent inflammation. Moreover, MDSCs can promote the differentiation of resting T cells to immunosuppressive Tregs [53, 54]. Tregs are a heterogeneous group of immunosuppressive T cells, e.g., the thymic Tregs (tTreg) maintain self-tolerance, whereas inducible Tregs (iTreg) prevent excessive inflammation. There are also tissue-resident, specific Treg populations, e.g., in the skin [55] and the adipose tissue [56]. The Forkhead box P3 (FoxP3) protein is the epigenetically-regulated master protein of the Tregs. Tregs are crucial immunosuppressive cells since they suppress the functions of effector T and B cells, inhibit the activity of natural killer (NK) cells, and promote the formation of other immunosuppressive cells, e.g., tolerogenic dendritic cells (DC) and anti-inflammatory M2 macrophages [57–59].

The host cells in the inflammatory microenvironment are able to educate immune cells, e.g., to switch pro-inflammatory cells to immunosuppressive phenotypes [60]. For instance, pro-inflammatory macrophages, called M1 macrophages, can be polarized to immunosuppressive M2 phenotypes in chronic inflammation [52]. The tumor microenvironment with persistent inflammation contains immunosuppressive tumor-associated macrophages (TAM) which suppress the antitumor activity of effector immune cells [61]. The immunosuppressive network also includes regulatory subsets of B cells (Breg) [62], DC cells (DCreg) [63], NK cells (NKreg) [64], and type II NKT cells [65] (Fig. 1). These immunosuppressive subsets of each immune cell type are heterogenous with respect to their phenotypes and functions and in addition, there exists a clear tissue-specificity in their properties. These regulatory cells have not been as extensively studied as MDSCs, Tregs, and M2 subsets of macrophages. Recently, I have reviewed the common properties of each member of immunosuppressive network and discussed how their changes are associated with the aging process [59]. Immunosuppressive cells possess a diverse armament of tools with which they not only regulate the immunosuppressive potency of the network but also suppress the functions of effector immune cells, e.g., the properties of T, B, DC, and NK cells. Common immunosuppressive mechanisms include (i) secretion of anti-inflammatory cytokines, such as TGF-β, IL-4, IL-10, IL-1 receptor antagonist (IL-1ra), (ii) release of reactive oxygen and nitrogen species (ROS/RNS), (iii) secretion of immunosuppressive compounds like adenosine and prostaglandin E2 (PGE2), (iv) expression of inhibitory immune checkpoint receptors, e.g., programmed cell death protein-1 (PD-1)/PD-L1 and cytotoxic T-lymphocyte associated protein 4 (CTLA4), and (v) expression of enzymes catabolising amino acids, such as indoleamine 2,3-dioxygenase 1 (IDO1) and arginase 1 (ARG1) [4, 66–68]. The remodelling of immune cells toward either an immunosuppressive or an immunosenescent state is underpinned by epigenetic regulation [69, 70].

### Immunosuppressive activity induces senescence state in immune and non-immune cells

The purpose of immunosuppression is to prevent excessive inflammatory responses although recent observations have indicated that the immunosuppressive period can be extended to the post-resolution phase since immunosuppressive cells can support the repair processes of host tissues [71, 72]. Indeed, the suppressive armament exploited by immunosuppressive cells (see above) contains many tools, e.g., secretion of IL-10, TGF-β, PGE2, and ROS/RNS as well as the depletion of distinct amino acids, which are not only drivers of immunosenescence but they can also enhance the senescence of non-immune cells [4, 5]. Senescent immune cells display a reduced ability to function as active effector cells which is attributed to the remodeling process in the immune system, especially in persistent inflammatory states [5, 73–76] (Fig. 1). Interestingly, immunosuppressive cells can induce changes in the phenotypes of immune cells which are very similar to those of immunosenescent cells. For instance, Tregs can induce the senescent phenotype of T cells [76]. Moreover, M-MDSCs and Tregs reduce the efficacy of immunosurveillance and the cytotoxic activities of NK and CD8 T cells [77–79]. For instance, an exposure to TGF-β suppressed the cytotoxic activity of human NK and CD8 T cells by inhibiting the expression of natural killer group 2D (NKG2D) receptor [80]. Nagaraj et al. [81] demonstrated that MDSCs were able to nitrate the TCR complex and thus inhibiting the antigen recognition of mouse tolerant CD8 T cells. Furthermore, MDSCs can impair the properties of DCs and B cells [74, 82]. In fact, immunosenescent cells display many of the characteristics encountered in the senescence of non-immune cells, e.g., telomere shortening, DNA damages, augmented expression of SA-β-Gal and cell cycle inhibitors (p16INK4a, p21WAF1, p53) as well as increased oxidative and endoplasmic reticulum stresses [5, 76, 83, 84]. Immunosenescence is not only associated with the aging process but it commonly occurs in chronic inflammatory states, especially those associated with the aging process [85, 86]. The accumulation of immunosenescent cells also occurs in chronic infections and autoimmune diseases, such as rheumatoid arthritis. [87, 88].

As discussed above, inflammatory mediators evoke a counteracting immunosuppression which impairs the function of NK and CD8 T cells. Consequently, the surveillance of senescent cells is inhibited because NK cells and CD8 T cells are the major surveying cells safeguarding tissue homeostasis. This impaired immune surveillance leads to an accumulation of senescent cells in both the immune system and peripheral tissues [89]. It is known that NK cells can recognize senescent fibroblasts and eliminate them through perforin-mediated cytolysis [90]. Ovadya et al. [89] demonstrated that the ablation of the *Perforin 1 (Prf1)* gene in mice increased the accumulation of senescent cells within tissues, triggered chronic inflammatory state, and finally reduced the survival of the animals. The NKG2D receptors of NK and CD8 T cells recognize the NKG2D ligands, especially the increased levels of MHC class I polypeptide-related sequence A (MICA) and UL16-binding protein 2 (ULBP2) proteins, on the surface of senescent cells and subsequently trigger their clearance [91]. However, inflammaging and chronic inflammatory states can impair the function of the NKG2D/NKG2D ligand axis and thus impair the cytotoxicity of NK and CD8 T cells. For instance, immunosuppressive cells, e.g., MDSCs, Tregs, and M2 macrophages, can inhibit the expression of NKG2D in NK cells through membrane-bound TGF-β1 signaling or the IDO1-mediated kynurenine secretion [92, 93]. Recently, I reviewed the mechanisms which immunosuppressive cells exploit to inhibit the surveillance and clearance of senescent cells [94]. I proposed a feed-forward model in which chronic low-grade inflammation induced a compensatory immunosuppression which subsequently enhanced the accumulation of pro-inflammatory senescent cells. It is well known that senescent cells, both immune and non-immune cells, secrete pro-inflammatory mediators and thus they are able to propagate an inflammatory state both during aging and in chronic inflammatory states [3, 5, 95].

### Immunosuppressive activity increases with aging

The aging process is associated with a remodeling of the immune system, both innate and adaptive immunity. It seems that the chronic inflammaging state triggers the remodelling process stimulating a counteracting immunosuppression which leads to immunosenescence (Fig. 1). This pathological highway is well known in many chronic inflammatory states (see below). Currently, although inflammaging and immunosenescence have been widely recognized as the driving forces behind the aging process, these two processes are not able to explain many clinical observations without the inclusion of an immunosuppressive component (see below). There is convincing evidence that inflammaging is associated with increased immunosuppressive activity, as also occurs in other chronic inflammatory conditions. For instance, inflammaging enhanced myelopoiesis in the bone marrow [96], a process which was associated with an age-related increase in the level of immunosuppressive MDSCs in the circulation of mice [97] and humans [98]. Accordingly, the number of Tregs was augmented with aging in human blood [78, 99]. Studies on the bone marrow (BM) and secondary lymphoid organs have revealed that in these tissues the upregulation in the numbers of MDSCs and Tregs with aging was more robust than in the circulation. For instance, Flores et al. [100] demonstrated that the percentage of MDSCs robustly increased with aging in both mouse BM and spleen. There are numerous reports which have revealed that the presence of MDSCs significantly increased with aging in mouse spleen and lymph nodes [97, 101]. Correspondingly, the level of Tregs was also augmented with aging in the spleen and lymph nodes [99, 102]. However, there exist different subsets of Tregs, e.g., thymic (tTreg), peripheral (pTreg), and inducible (iTreg), which have some similar and some distinctive properties [103]. It is known that the levels of natural FoxP3^+^ Tregs (nTreg), originated either from CD4 T or CD8 T cells, increase with aging, whereas those of iTreg and tTreg decline during the aging process [104, 105]. The decrease in the numbers of tTreg cells was most probably associated with an age-related involution of the thymus. However, Szurek et al. [106] demonstrated that the markers of tTreg and iTreg, i.e., helios and neuropilin-1 (Nrp-1), did not distinguish tTreg cells from iTregs in mice. Interestingly, van der Geest et al. [107] revealed that the proportion of the naïve Tregs declined with aging in human blood, whereas that of the memory Tregs (memTreg) clearly increased. Moreover, the level of memTregs inversely correlated to the human vaccination efficiency [107]. In conclusion, it seems that the remodeling of the immune system involves a significant increase in the accumulation of immunosuppressive MDSCs and Tregs.

There are some technical problems to quantify whether the presence of MDSCs and Tregs increases with aging in non-immune tissues. However, there are observations indicating that the level of MDSCs increased with aging in mouse and human skin [108]. Ruhland et al. [108] reported that the numbers of CD14, CD15, and CD33-positive cells were robustly increased in the skin of elderly people. These proteins are common markers of human MDSCs. They also demonstrated that stromal senescence enhanced the occurrence of both MDSCs and Tregs within mouse skin. The role of Tregs in the aging process is still far from clear since tissues contain several unique populations of tissue-resident Tregs which are crucially involved in the regulation of tissue homeostasis [58, 109]. For instance, the Tregs found in mouse adipose tissue are involved in the regulation of energy metabolism and insulin resistance. The numbers of Treg cells significantly increased with aging in the visceral adipose tissue of lean mice, whereas obesity induced a decline in the numbers of Tregs [110, 111]. However, Bapat et al. [112] demonstrated that the depletion of fat-resident Tregs prevented the development of age-associated insulin resistance (IR). It is known that there exist two types of insulin resistance, i.e., the age-related IR and the obesity-associated IR. Given that obesity increases inflammation, it seems that the obesity-associated IR was induced by the tissue-infiltrated Tregs.

In addition to MDSCs and Tregs, there are clear changes with aging in the phenotypes of other members of the immunosuppressive network [59]. For instance, tissue-resident macrophages displayed an increased polarization toward the anti-inflammatory M2 phenotype not only in BM, spleen, and lymph nodes but also in lungs and skeletal muscles [113, 114]. However, because macrophages are remarkably plastic cells, they can adapt to local disturbances in their microenvironment and thus express the M1 phenotype. Nonetheless, Duong et al. [115] demonstrated that the depletion of macrophages in elderly mice augmented the antitumor activity of T cells and improved the responses of mice to tumor immunotherapy. These observations indicate that macrophages can augment their immunosuppressive activity during the aging process. There is convincing evidence that both MDSCs and Tregs in aged mice/humans are immunosuppressive, i.e., they suppress the antigen-induced proliferation of T cells, increase the production of IL-10, TGF-β, and ROS, inhibit the maturation and function of DCs, decrease the cytotoxic activity of NK and CD8 T cells and increase the susceptibility to infections and cancer formation [78, 97, 101, 102]. It seems that the low-grade inflammation associated with aging is a driving force for immunosuppression and subsequent immunosenescence.

### Increased immunosuppression is associated with chronic inflammatory states

Systemic chronic inflammation has a major role in the pathogenesis of many chronic diseases, e.g., cardiovascular diseases (CVD), COPD, CKD, hepatic steatosis, many cancers, autoimmune diseases, and neurodegenerative diseases [6]. There is a multitude of causes for chronic inflammatory conditions; many of these are still unknown such as that of the aging process. Nonetheless, a chronic inflammatory state activates immunosuppressive cells which impair the functional abilities of the immune system, thus promoting immunosenescence (Fig. 1). Studies conducted on cardiovascular diseases, e.g., atherosclerosis, hypertension, acute coronary syndrome, and stroke, have indicated that immunosuppressive cells were involved in the pathology of CVD [116–119]. For instance, the occurrence of a stroke increased the level of Tregs which attenuated inflammatory responses and enhanced the appearance of post-stroke regeneration [119, 120]. Accordingly, stroke increased the level of circulating M-MDSCs in human stroke patients [121]. It is known that stroke increases the risk for post-stroke infections, probably due to immunosenescence (see below). In COPD patients, the number of circulating MDSCs significantly increased [122], whereas the Th17/Treg balance was upregulated in the acute phase of COPD but significantly decreased when COPD moved to the stable phase [123]. This indicated that there existed an immunosuppressive state in the stable COPD. Chronic inflammation enhanced the progression of non-alcoholic fatty liver disease (NAFLD) which is a world-wide public health problem [124]. Zhou et al. [125] demonstrated that the accumulation of MDSCs into human liver during the progression of NAFLD positively correlated with the clinical parameters of liver disease. There is clear evidence that the balance between Th17 and Treg cells has a significant role in the progression of NAFLD in both mice and humans [126, 127]. An increased level of pro-inflammatory Th17 cells enhanced the progression of NAFLD, whereas Tregs prevented the generation of NAFLD.

Many persistent viral infections, e.g., HIV and herpes simplex virus (HSV), stimulate a chronic low-grade inflammatory state which is associated with increased immunosuppression and immunosenescence [128–130]. More severe, pathogen-induced sepsis also triggers both pro-inflammatory and immunosuppressive phases [131, 132]. It seems that MDSCs, especially M-MDSCs, have a key role in the resolution of inflammation in infectious foci induced by bacteria or viruses. In non-resolving infections, MDSCs stimulate the immunosuppressive microenvironment which prevents excessive inflammation [128]. For instance, Sarkar et al. [129] demonstrated that in mouse ocular HSV1 infection, MDSCs suppressed the proliferation and cytokine production of activated CD4 T cells, i.e., MDSCs induced the immunosenescence of CD4 T cells. They also reported that an adoptive transfer of the in vitro-generated MDSCs into infected mice expanded the pool of Tregs and subsequently reduced the severity of the HSV1-induced ocular infection. Currently, there are observations that COVID-19 infection induced a massive expansion of PMN-MDSCs which inhibited the specific T cell responses and increased the fatal outcome in patients infected by COVID-19 [133]. Accordingly, the number of Tregs and the expression level of FoxP3 protein were robustly accentuated in the blood of COVID-19 patients and these changes closely correlated with the severity of the disease [134]. The robust increase in the levels of MDSCs and Tregs in the COVID-19 syndrome indicates that overwhelming inflammation activates immunosuppressive cells which induce immunosenescence and an inefficient immune defence. It seems that the aging-induced immunosuppressive changes leading to immunosenescence in the immune system enhance the severity of the COVID-19 syndrome in older patients [135].

Chronic inflammation is an important player in different phases of tumorigenesis [10]. Inflammation is able to predispose to the development of tumors and subsequently the inflammatory tumor microenvironment (TME) enhances the formation of local immunosuppression [82, 136]. The immunosuppressive state is generated by the presence of both invading and resident immunosuppressive cells, e.g., TAMs, MDSCs, Tregs, and cancer-associated fibroblasts (CAF) [136–138]. Immunosuppression induces an immunosenescent state in TME by inhibiting the normal functions of macrophages, T cells, NK cells, and dendritic cells [139]. This local immunosenescence allows tumor cells to evade immune surveillance. The inflammatory state not only triggers the senescence of immune cells but it also elicits cellular senescence in non-immune cells which secrete pro-inflammatory mediators (SASP state), thus aggravating the inflammatory state in TME [140]. This kind of immunosuppression-induced immune senescence poses a challenge for cancer immunotherapies. Given that chronic inflammation, immunosuppression, and immunosenescence are involved in the aging process (see above), it has been claimed that an increased risk for tumors with aging could be attributed to an inflammaging process [141].

Photoaging is another local chronic process in which repeated UV-induced inflammatory insults enhance immunosuppression in the skin and consequently accelerate its aging process [142–144]. Skin exposure to UVR not only enhances photoaging but can also make the affected skin susceptible to carcinogenesis [145]. UVR induces both the local and systemic attenuation of the immune system [146, 147]. It has been reported that the induction of Tregs has a key role in the generation of UV-induced immunosuppression [148, 149]. The presence of tissue-resident Tregs is enriched in normal skin involving a heterogeneous population of Treg cells [150]. Treg cells have important functions in the skin, e.g., they augment wound healing, participate in hair follicle regeneration, prevent autoimmunity, and maintain immune tolerance against skin commensal microbes. It seems that there are different mechanisms involved in the activation of Tregs by UVR. For example, Soontrapa et al. [146] demonstrated that the UVB-induced stimulation of Tregs was mediated by PGE2 via signaling through the EP4 receptors in mouse epidermis. Moreover, Navid et al. [151] revealed that UVR stimulated aryl hydrocarbon receptor (AhR) signaling which triggered the Treg-mediated immunosuppression in mouse skin. It is known that UVR promotes the catabolic breakdown of tryptophan to kynurenine and 6-formylindolo [3,2-b]-carbazole (FICZ) which are potent activators of AhR signaling and subsequently they stimulate FoxP3 expression and the activation of Tregs [152, 153]. Several studies have revealed that an increased expression of AhR factor in skin also predisposed to the development of skin cancers [154]. Photoaging is an interesting example of how the immunosuppression induced by repeated inflammatory insults not only accelerates the aging process but is also a risk factor for the development of skin cancers.

### Clinical disadvantages of age-related increase of immunosuppression

As stated above, the aging process increases the risk for cancer development and it aggravates infections and several inflammatory diseases. On the other hand, many cancers and chronic inflammatory conditions accelerate the aging process (see above). It seems that inflammation and the subsequent immunosuppression, triggered either by the aging process or tumors, possess similar properties which mutually can promote each other’s pathology. The immunosenescence encountered in both states provides an escape of senescent cells and tumor cells from immune surveillance by NK and CD8 T cells. For instance, Ruhland et al. [108] demonstrated that an experimentally induced senescence of stromal cells in mouse skin increased inflammation and established an immunosuppressive microenvironment which promoted tumorigenesis. The SASP state of senescent cells increased the secretion of IL-6 which triggered the expansion of MDSCs and Tregs in mouse skin. There is convincing evidence that the inflammation-induced recruitment and expansion of MDSCs and Tregs promote the age-related tumor incidence in different tissues [101, 155, 156]. It has been recognized that chronic inflammation and immunosuppression shape the chromatin landscape which might modify the genetic background not only during the aging process but in many other diseases [157, 158]. Interestingly, many investigators have reported that the epigenetic aging profile (see above) predicts the risk of cancer, cardiovascular disease, and mortality in many diseases [159, 160]. Currently, the molecular significance of the aging-associated epigenetic drift in tumorigenesis needs to be clarified.

Aged people are not only susceptible to tumorigenesis but also infections induced by diverse pathogens. There is convincing evidence that the age-related increase in immunosenescence is a major cause of an increased sensitivity to infections in the elderly [135, 161, 162]. An increased susceptibility to infections has also been observed in individuals suffering from many chronic inflammatory diseases and immunocompromised states. These conditions display an enhanced immunosenescence although there are differences in the decline in immune defence. As discussed above, infections are associated with an increased activity of immunosuppressive cells which evoke immunosenescence of effector immune cells (Fig. 1). For instance, the age-related immunosenescence affects infections since it (i) induces defects in the functions of neutrophils, (ii) decreases the responses of T cells, e.g., the affinity of TCR for antigens and the cytotoxicity of CD8 T cells, (iii) diminishes the maturation and antigen presentation by DCs, (iv) disturbs the functions of B cells, (v) down-regulates the activating receptors of NK cells [161, 163–165]. The age-related decline in immune host defence increases a person’s susceptibility to infections as well as promoting the persistence of infections. Currently, it seems that cytokines secreted by immunosuppressive cells, e.g., IL-10 and TGF-β, are important epigenetic regulators which suppress the functions of effector immune cells by modifying their chromatin landscape [5, 166, 167].

It is known that vaccination efficiency decreases with aging. This decline has been attributed to the age-related immune deficiency, i.e., immunosenescence [161, 164, 168]. Vaccination efficiency is reduced in cancers and chronic inflammatory states, such as obesity [169], indicating that immunosenescence is involved. In addition, chronic CMV infection reduces the responsiveness to influenza vaccination in aged people [170]. The decline in vaccination efficacy has been observed in different vaccination protocols involving influenza and cancer vaccines. There is convincing evidence that the decrease in vaccination efficiency with aging is caused by an increased level of immunosuppressive cells, e.g., MDSCs and Tregs [169, 171, 172]. Corsini et al. [171] demonstrated that an increased IL-10 level in the elderly was associated with a low antibody response to influenza vaccination. Moreover, a decrease in the level of MDSCs was reported to improve the vaccination efficacy [173]. Currently, there are several strategies aiming to improve vaccination efficiency in elderly people. For instance, attenuating immunosenescence via the inhibition of immunosuppression might be a successful approach.

There are many studies indicating that the efficiency of immunotherapeutic treatments becomes diminished in elderly people [174]. The senescence of the immune system is a major difficulty in the activating immunotherapies, e.g., in the treatments of cancer patients [175, 176]. Given that aging and cancers increase immunosuppression and subsequently promote immunosenescence (Fig. 1), there are different approaches to inhibit the functions of MDSCs and Tregs [177, 178]. It is known that MDSCs mount a major resistance to immune checkpoint inhibitor (ICI) therapies [179]. The clinical efficiency of ICI treatments can be improved by the combination therapies which involve the chemical inhibition of MDSC function. For instance, the combination of gemtuzumab ozogamicin (GO), an immunotoxic conjugate linking CD33 antibody with cytotoxic calicheamicin, has been exploited to target and eliminate MDSCs in human cancer patients [180]. Fultang et al. [180] demonstrated that in the treatment of acute myeloid leukemia, the GO therapy targeted MDSCs and also improved the chimeric antigen receptor T cell (CAR-T) immunotherapy. Moreover, tumor immunosuppression can be attenuated and immunotherapy enhanced by using STAT3 inhibitors [181], all-trans retinoic acid (ATRA) [182], and many phytochemicals [183]. The provision of Treg immunotherapy is a more difficult challenge since Tregs maintain immunological tolerance and immune homeostasis. However, there are some specific surface molecules on Treg cells which can be targeted in cancer immunotherapy [178]. Hurez et al. [184] demonstrated that the depletion of MDSCs improved antitumor immunity in aged mice, whereas a reduction in the numbers of Tregs increased the level of MDSCs in old mice. They also revealed that the exhaustion of MDSCs was an effective treatment in aged but not in young mice bearing B16 tumors. However, combining the depletions of both MDSCs and Tregs improved the therapeutic efficacy in old B16-bearing mice [184]. These few examples clearly indicate that an increased level of immunosuppressive activity is a real problem in exploiting immunotherapy in the elderly.

Increased immunosuppression with aging not only induces immune deficiency but it also disturbs the homeostasis of the host tissues. Immunosuppressive cells possess a molecular armament which inhibits effector immune cells, e.g., anti-inflammatory cytokines, ROS/RNS, and enzymes catabolizing amino acids (see above). These mechanisms also affect the neighboring non-immune cells in host tissues [4]. For instance, TGF-β signaling is able to induce cellular senescence and tissue fibrosis as well as evoking a remodelling of the extracellular matrix (ECM) [185–187]. An increase in the level of TGF-β signaling has been associated with many age-related diseases, such as muscle and skin atrophies and cardiovascular diseases. Accordingly, IL-10 signaling can inhibit autophagy [188] which is known to decline with aging. IL-10 and TGF-β cytokines can also modify the chromatin landscape via the STAT3 and SMAD pathways and thus induce epigenetic changes in gene expression profiles with aging. The secretion of ROS/RNS by immunosuppressive cells is an important mechanism to inhibit the activity of effector immune cells and thus generate immunosenescence [189]. Many researchers believe that ROS and oxidative stress play a crucial role in tissue degeneration involved in the aging process. In addition, immunosuppressive cells robustly express ARG1 and IDO1 enzymes which catabolize L-arginine (L-Arg) and L-tryptophan (L-Trp), respectively [67]. Since there is an increase in the activities of ARG1 and IDO1 in inflammatory conditions, this will deplete L-Arg and L-Trp amino acids and thus not only suppress the proliferation of immune cells but also inhibit the protein synthesis in neighboring cells, thus enhancing tissue atrophy [4]. Tissue atrophy is typically encountered in both the aging process and chronic inflammatory diseases.

### Clinical benefits of the age-related increase of immunosuppression

Increased immunosuppression and subsequent immunosenescence have not only harmful effects but there are studies indicating that age-related immune deficiency can confer some benefits, e.g., transplantation tolerance is enhanced with aging [190–193]. It is known that the age of donor and recipient patients determines the outcome of tissue transplantation [194]. There is a common rule indicating that old persons are poor donors but good receivers. However, it is dependent on the tissue being transferred since for instance, kidney transplantation requires a younger donor than that of heart transfer [194]. The immune senescence of the recipient patient improves the acceptance of transplants of different organs [190, 195, 196]. The better tolerance of transplants in older recipients is attributed to the age-related increase in the immunosuppressive activity which inhibits the functions of effector immune cells, such as the surveillance by NK and CD8 T cells (see above). There are many studies indicating that the transplantation tolerance is induced by the expansion of immunosuppressive regulatory myeloid cells, i.e., MDSCs, Tregs, DCregs, and M2 macrophages [192, 193, 197]. In proof of principle, adoptive cell therapies with immunosuppressive cells have significantly improved transplantation tolerance [198].

Immunosenescence is associated with many autoimmune diseases, e.g., rheumatoid arthritis and multiple sclerosis [87, 199]. With respect to the role of aging, there are significant differences between the diverse autoimmune diseases. However, it seems that the incidence of autoimmune diseases increases with aging although the severity of diseases can be reduced [200, 201]. On the other hand, some autoimmune diseases, e.g., rheumatoid arthritis, accelerate the aging process, similarly to the situation in many other chronic inflammatory diseases [43, 200]. There is substantial evidence that MDSCs have a crucial protective role in autoimmune diseases [202–204]. Given that MDSCs suppress T cell responses and the activity of NK cells, it is not surprising that MDSCs are able to enhance immune tolerance in autoimmune diseases. However, there are many questions on their specificity in different tissues and diseases, especially related to the function of separate subpopulations of MDSCs [204]. It seems that PMN-MDSCs are more immune suppressive than M-MDSCs, whereas M-MDSCs promote the development of Tregs and inhibit the functions of B cells, e.g., in autoimmune arthritis. Treg cells maintain self-tolerance and thus disturbances in their activity are thought to lead to the development of autoimmune diseases [205]. There are a variety of approaches, either drug-based or cell-based therapies, targeting both MDSCs and Tregs aiming to enhance their immunosuppressive properties in autoimmune diseases [204–206]. Currently, adoptive cell therapies exploiting engineering technologies in the production of the antigen-specific Tregs and chimeric antigen receptor (CAR) Tregs have been developed to induce specific immune tolerance in autoimmune diseases [206, 207]. It does seem that the age-related increase in immunosuppressive activity is able to reduce the severity of autoimmune diseases but its level and specificity are insufficient to totally resolve the disease.

## Conclusions

Chronic inflammatory states stimulate a counteracting immunosuppression which promotes immunosenescence in an attempt to protect tissues against excessive and detrimental inflammatory processes. There is convincing evidence that the age-related chronic low-grade inflammation also induces immunosuppression which consequently enhances the senescence of the immune system. Interestingly, there are observations that the aging process increases the risk for cancers as well as augmenting the risk for suffering chronic inflammatory diseases and conversely, repeated or persistent inflammatory states accelerate the aging process. This means that inflammatory components are a major driving force in the aging process. On the other hand, an age-related increase in immunosuppressive activity and immunosenescence explain many clinical consequences associated with aging. For instance, the incidence of cancers increases with aging since immunosuppression has a crucial role in tumorigenesis. Moreover, elderly people are susceptible to long-term infections, i.e., increased immunosuppression inhibits the function of effector immune cells and thus the resolution of infections tends to be prolonged in the elderly. It is also known that there are declines in both vaccination efficiency and the efficacy of immunotherapies in aged people. The senescent immune system, associated with either aging, cancers, or chronic inflammatory diseases, is unable to respond effectively to vaccines and antibodies. Currently, there are trials in progress with many drug- or cell-based therapies attempting to reinforce the inefficient immune system not only in cancer and chronic inflammatory states but also in the aging process.
